# Using a Geographical-Information-System-Based Decision Support to Enhance Malaria Vector Control in Zambia

**DOI:** 10.1155/2012/363520

**Published:** 2012-04-02

**Authors:** Emmanuel Chanda, Victor Munyongwe Mukonka, David Mthembu, Mulakwa Kamuliwo, Sarel Coetzer, Cecilia Jill Shinondo

**Affiliations:** ^1^National Malaria Control Centre, Ministry of Health, P.O. Box 32509, Lusaka, Zambia; ^2^Ministry of Health, Headquarters, Ndeke House., P.O. Box 30205, Lusaka, Zambia; ^3^Malaria Research Programme, Medical Research Council, Ridge Road, Durban, South Africa; ^4^School of Medicine, University of Zambia, P.O. Box 50110, Lusaka, Zambia

## Abstract

Geographic information systems (GISs) with emerging technologies are being harnessed for studying spatial patterns in vector-borne diseases to reduce transmission. To implement effective vector control, increased knowledge on interactions of epidemiological and entomological malaria transmission determinants in the assessment of impact of interventions is critical. This requires availability of relevant spatial and attribute data to support malaria surveillance, monitoring, and evaluation. Monitoring the impact of vector control through a GIS-based decision support (DSS) has revealed spatial relative change in prevalence of infection and vector susceptibility to insecticides and has enabled measurement of spatial heterogeneity of trend or impact. The revealed trends and interrelationships have allowed the identification of areas with reduced parasitaemia and increased insecticide resistance thus demonstrating the impact of resistance on vector control. The GIS-based DSS provides opportunity for rational policy formulation and cost-effective utilization of limited resources for enhanced malaria vector control.

## 1. Introduction

In Sub-Saharan Africa, malaria remains a major cause of morbidity and mortality [1]. Its transmission is driven by a complex interaction of the vector, host, parasite, and the environment, and is governed by different ecological and social determinants [2, 3]. The survival and bionomics of malaria vectors are affected by climate variability, that is, rainfall, temperature, and relative humidity [4]. In this light, even minute spatial variations and temporal heterogeneities in the mosquito population can result in significant malaria-risk [5, 6] and its endemicity [7–9].

Since malaria distribution is not homogeneous, much effort needs to be expended towards defining local spatial distribution of the disease [2] precedent to deployment of interventions [10]. In resource constrained environments, monitoring, and evaluation is often incomprehensive and irregular and tend to lack the actual spatial and temporal distribution patterns. If transmission determining parameters are to be harnessed effectively for decision-making and objectively plan, implement, monitor, and evaluate viable options for malaria vector control [11], they must be well organized, analyzed, and managed in the context of a geographical-information-system- (GIS-) based decision support system (DSS) [3, 12].

While vector control interventions are being deployed according to the World Health Organization-led Integrated Vector Management Straandtegy [10, 13, 14], prompt availability of relevant spatial and attribute data is vital to support malaria surveillance, management research, and policy initiatives. Different strategies coupled with new technologies such as mapping, GIS, and DSS, and spatial and temporal modelling are being harnessed to more effectively target limited surveillance, prevention, and control at research scale [15]. However, potential utilization of these approaches and their incorporation in the operational malaria vector control programmes remains a significant constraint and continues to receive limited attention [16, 17].

Until recently, very few malaria endemic countries had incorporated the GIS technology into operational malaria control programmes, that is, in South Africa and the Lubombo Spatial Development in Mozambique in Southern Africa, where it has been harnessed for case mapping and monitoring of vector control coverage [18, 19]. In India, it has been used to monitor malaria transmission attributes as well as social-economical and social cultural aspects of malaria [3]. To achieve enhanced utilization of mapping and GIS technologies in operational malaria control, sharing of experiences with GIS and emerging technologies by malaria control programmes is critical [15, 17]. Herein is provided a review of data related to the operational use of a GIS-based DSS [12, 20] for optimal deployment, monitoring, and evaluation of entomological interventions for malaria control in Zambia.

## 2. Materials and Methods

The integration of operational and logistical data for malaria control program planning with epidemiological data will serve to strengthen both the epidemiological analysis and the planning and execution of control programs. GIS facilitate the integration of quantitative malaria determination and control data with data obtained from maps, satellite images, and aerial photos. A comprehensive review of data collected through nationally representative malaria indicator surveys and insecticide resistance data in major malaria vectors: *An. gambiae s.s*, *An. arabiensis*, and *An. funestus*, including the comparative impact of main stream vector control interventions, has been conducted in Zambia.

### 2.1. Intervention

The intervention consists of scaled-up indoor residual spraying (IRS) in urban and periurban areas and insecticide treated nets (ITNs) in rural areas [14, 21–23]. Indoor residual spraying is implemented through annual campaigns with 85% coverage of eligible households using pyrethroids at 25 mg/m^2^ (Syngenta and Bayer) and DDT at 2 g/m^2^ (Avima) at the beginning of the peak malaria transmission period [24]. Pyrethroid-impregnated ITNs, that is, PermaNet (Verstargaard frandsen) and Olyset (Sumitomo Corporation), are deployed through antenatal and child clinics, equity programme, community mass distribution, and commercial sector and strive towards attaining 100% coverage in eligible areas [25]. This effort is coupled with effective case management by provision of definitive diagnosis, using rapid diagnostic tests (RDTs) and microscopy, and treatment with artemisinin-based combination therapy (ACT), and intermittent preventive treatment (IPT) to expecting mothers [21]. This is further augmented with interactive information, education, and communication (IEC) and behavioural change and communication (BCC) strategies to enhance utilization of interventions [26]. There is strong operations research feeding into and guiding implementation.

### 2.2. Spatial Decision Support System

**Figure 1:**
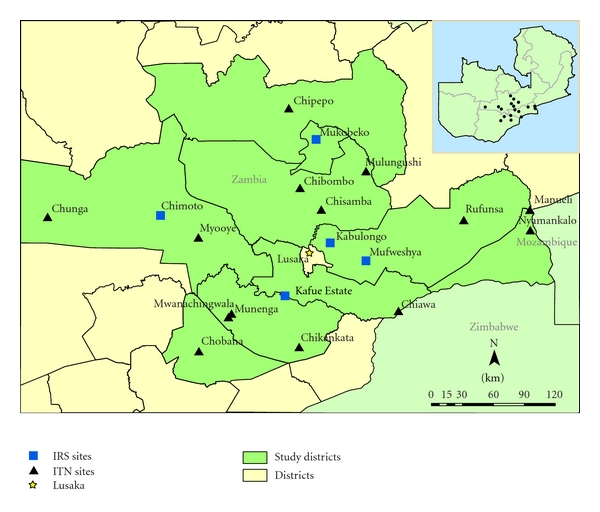
Map of Zambia showing the location and spatial distribution of GIS-based decision support system monitoring sentinel sites.

Zambia is situated in the Southern African region with a population of approximately 12 million, 45% of whom are below the age of fifteen [27]. Malaria is endemic country-wide and transmission is throughout the year with peak in rain season. The disease is the leading cause of morbidity and mortality accounting for 40% of outpatient attendances, 45% of hospital admissions with 47% and 50% of disease burden among pregnant women, and children under five years of age, respectively. Current trends in the country indicate that malaria is responsible for at least 3 million clinical cases and about 6,000 recorded deaths annually, including up to 40% of the under five deaths and 20% of maternal mortality [28, 29]. Malaria stratification aids in the development of community-based malaria control programs, by accumulating past experiences with and solutions to different factors associated with malaria outbreaks. Stratification can also point to the existing inequalities in resources, allowing for a more equal and homogeneous distribution of available resources [30]. In this regard, to allow for adaptation of intervention policy, procedures and methods to better outcomes, nineteen GIS-based sentinel sites, distributed amongst nine districts within a 350 km radius of the capital Lusaka (Figure 1), were established for the continual monitoring and collation of key malaria data such as parasitaemia risk, insecticide resistance profiles in vectors and impact of interventions on malaria prevalence. The study region is characterized by reduced seasonality of transmission with extensive vector control through IRS at 6 sites and ITNs in all sites from 2003 to 2010 by the National Malaria Control Programme (Figure 1). 

### 2.3. Spatial Monitoring of Interventions

The spatial and temporal impact of IRS and ITNs on human parasite prevalence and insecticide resistance status in major malaria vectors was monitored. At each sentinel site annual household surveys were carried out annually from 2008 to 2010 to measure *Plasmodium falciparum *prevalence in children aged 1 to 14 [31, 32]. In Zambia, three nationally representative malaria indicator surveys (MISs) were also conducted in children under five years of age in 2006, 2008, and 2010 [33]. The MIS have been used (i) to estimate an empirical high-resolution parasitological risk map in the country and (ii) to assess the relation between malaria interventions and parasitaemia risk [34]. By standard WHO protocol, spatiotemporal insecticide resistance profiles of major malaria vectors: *Anopheles gambiae s.s, An. arabiensis, *and *An. funestus *were determined at sentinel sites and were extended to other regions of the country [32, 35, 36]. More data on spatial distribution of insecticide resistance to bendiocarb (0.01%), DDT (4%), deltamethrin (0.05%), lambda-cyhalothrin (0.05%), malathion (5%), and permethrin (0.75%) have been collected by different partners and collated by the National Malaria Control Programme (Figures 4 and 5).

## 3. Results

### 3.1. Spatial Prevalence of Malaria Infection

**Figure 2:**
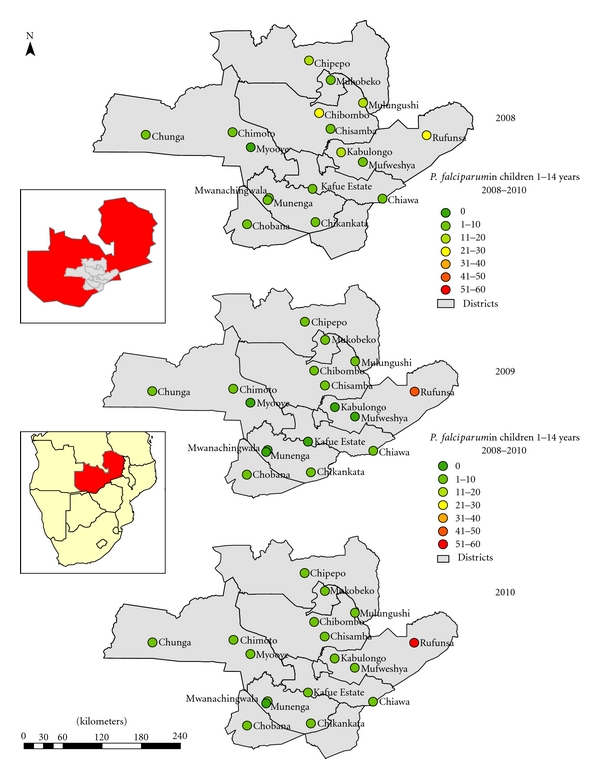
*P. falciparum* malaria parasite prevalence in children 1 to <15 years in monitoring sentinel sites from 2008 to 2010 surveys.

**Figure 3:**
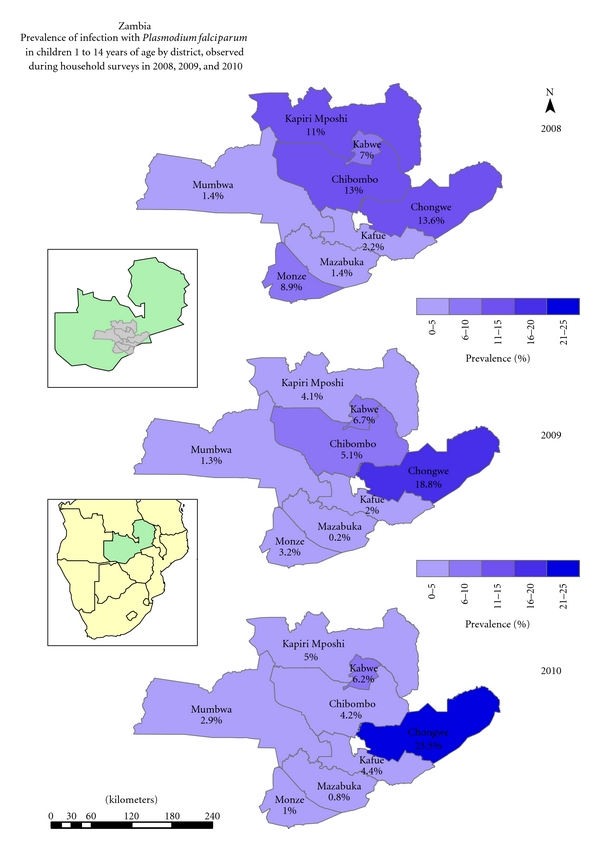
Prevalence of infection with *P. falciparum* in children 1 to <15 years as observed during the annual parasitaemia surveys from 2008 to 2010 by district.

*Plasmodium falciparum* accounts for 98% of all malaria infections in the country, causing the severest form of disease, with a low frequency of infections from *P. malariae* and *P. ovale, *and no transmission of *P. vivax. *The national malaria indicator survey for 2010 in children under the age of five years shows great spatial heterogeneity in prevalence of infection [37]. This has resulted in stratification of the country in three epidemiological categories: Type 1 areas with very low transmission and parasite prevalence of <1%, Type 2 areas with low transmission and prevalence of under 10%, and Type 3 areas with persistent high transmission and prevalence exceeding 20% at peak transmission season [33]. Cross-sectional surveys at sentinel sites (Type 2 areas) in children between 1 and 14 years across the study area (Figures 2 and 3) showed a combined prevalence of infection with *P. falciparum* to be below 10% albeit with great heterogeneity between IRS and ITN areas [32].

### 3.2. Spatial Distribution of Insecticide Resistance Profiles

**Figure 4:**
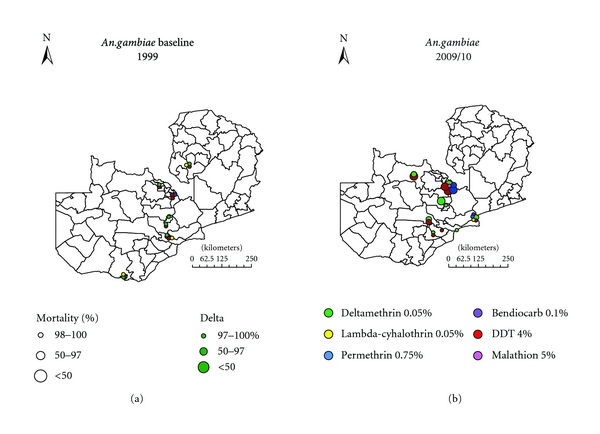
The spatial distribution of insecticide resistance in *An. gambiae s.l. *in 1999 compared to 2009/10 in Zambia.

**Figure 5:**
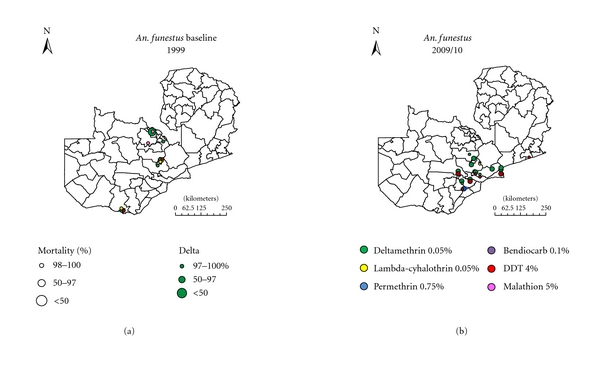
The spatial distribution of insecticide resistance in *An. funestus* in 1999 compared to 2009/10 in Zambia.

By standard WHO protocol, suspected and overt resistance to insecticides being harnessed for vector control, pyrethroids, and DDT, has been detected in all the key vectors in operational settings of both IRS and ITNs (Figures 4 and 5). High levels of insecticide resistance have been detected in both *An. gambiae s.l* and *An. funestus* to pyrethroids and DDT. There is great variation in the level of resistance between IRS and ITNs localities, with exceptionally higher level resistance being detected in IRS areas compared to ITNs areas (*P* < 0.0001). The west form of knockdown resistance (kdr) mutation has been detected in* An. gambiae s.s* in some areas of the country with crossresistance between pyrethroids and DDT [32].

### 3.3. Spatial Impact of Interventions on Malaria Prevalence

**Figure 6:**
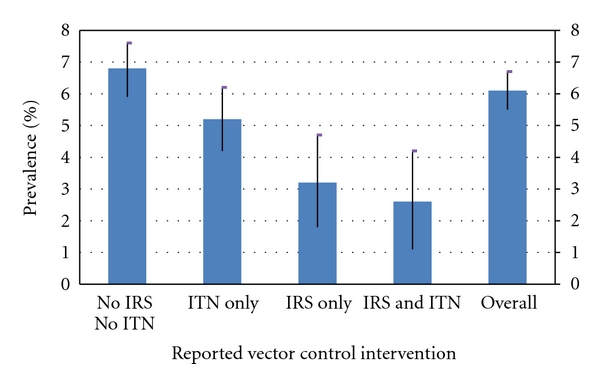
Prevalence of infection in children 1 to <15 years of age in Zambia by reported vector control intervention (2008, 2009, and 2010 combined). IRS: Indoor Residual Spraying; ITN: Insecticide Treated Net.

The overall prevalence of infection in children whose house had not been sprayed in the past year and did not sleep under a net the night before the survey was 6.8%. Children who slept under a net, but whose house had not been sprayed during the past year, had a prevalence of infection of 5.2%. Children whose house had been sprayed during the past year, but did not sleep under a net, had a significantly lower prevalence of infection of 3.2%. Children who slept under a net in a dwelling that had been sprayed had the lowest risk of infection with a prevalence of 2.6%. Thus incremental effect was observed for combined use of IRS and ITNs (Figure 6) [32].

## 4. Discussion

Given the spatial heterogeneity in the distribution of malaria vectors and variations in the inherent malaria risk, GIS has potential applications in deployment and monitoring of interventions. For resource-constrained malaria-endemic Sub-Saharan African countries, like Zambia, the need for a GIS-based malaria information system cannot be overemphasized. Until recently, decisions in the malaria control programmes were taken on an *ad hoc* basis driven by limited empirical evidence and undoubtedly resulting in misdirection of the limited resources available.

Following the increased funding for malaria control [38] particularly in Sub-Saharan Africa [39, 40], insecticide-based malaria vector control interventions are being scaled up in most endemic countries [41] albeit with limited empirical evidence on their impact and amenability to local settings. Invariable monitoring, evaluation, and continuous surveillance of vector species abundance, infectivity, insecticide resistance status, and parasite prevalence in the population are imperative to ensure effective deployment of interventions and optimal utilization of limited resources [42]. The GIS-based decision support system is proving to be an invaluable tool to optimize impact assessment of malaria control interventions and thus rationalize resource utilization [1].

The use of GIS in Zambia has enabled detection of spatial trends of parasite prevalence following extensive deployment of front line vector control interventions. Cross-sectional prevalence surveys show continuous prevalence increase in children from 2008 to 2010 in Chongwe district. In Kapiri mposhi, Mumbwa, Mazabuka and Kafue districts, prevalence dropped between 2008 and 2009 but increased in 2010. However, progressive reduction in malaria prevalence was detected in Monze, Kabwe, and Chibombo districts from 2008 through to 2010 (Figure 3).

The GIS has introduced new dimensions to the understanding, prediction, analysis, and dissemination of spatial relations between disease, time, and space [43, 44]. It allows the integration of geographical referenced data, together with local knowledge in relational databases to accurately display complex interactions in simple formats [3]. The use of these data sets in a GIS provides an opportunity to integrate up-to-date information, local knowledge, and historical trends in a manner that draws attention to areas of change-associated problems and options for action. This makes GIS a tool not only for data analysis, but also for information management and decision-making thus facilitating policy formulation [18].

There was great heterogeneity in prevalence of malaria at sentinel sites relative to detected insecticide resistance in malaria vectors. At Chibombo, prevalence has been reducing despite high pyrethroid resistance detected in *An. funestus*. At Myooye and Chimoto, ITN deploying sites with high *kdr* mediated crossresistance to pyrethroids and DDT in *An. gambiae s.s*, prevalence was reducing and remained at a low level across the three years. However, Rufunsa, another ITN deployment area with high pyrethroid resistance in *An. funestus,* exhibited constant increase in parasitaemia despite high coverage of ITNs (Figure 2). 

The usefulness of a GIS-based DSS for planning and managing control programmes is dependent on the availability of accurate and raw data on malaria transmission-related parameters. Monitoring and evaluation of malaria interventions and understanding of their true impact on disease burden is essential for measuring performance of a control programme. An effective system for monitoring and evaluation and continuous surveillance requires integration of spatially and temporally explicit data for entomological and epidemiological outcome indicators. This allows for identification of disease prevalence, planning of effective interventions, assessments of reduction of vector exposure and malaria burden resulting from implemented control measures. Continuous surveillance capturing real time data enables routine monitoring and evaluation of programme to demonstrate goals and impact on malaria burden. This is essential to increasing the efficiency and effectiveness of malaria control efforts [42]. The effective control of malaria requires programme managers to have access to the most up-to-date information on the disease in order to best direct interventions efforts against the vectors.

Effective implementation and monitoring and evaluation of malaria control interventions have resulted in redefinition of stratification of the country in three epidemiological zones for malaria transmission potential in Zambia [33, 45]. This necessitates appropriate targeting of interventions guided by entomological and epidemiological evidence of active malaria transmission. Although the quality of data collection and archiving kept on improving, most data bases have been vertical. An excel spread sheet contained ITN data base capturing quantities distributed by district and year. The IRS database captured quantities of commodities and equipment, and spraying coverage per district and year. The ITNs have been monitored through a two component system: (1) compilation of information on number of ITNs distributed and (2) tracking ITN coverage and/or ownership and utilization rates by householders. Since 2000, IRS has been monitored based on generic reporting forms for formal spraying management introduced by World Health Organization. This set comprised daily spray operator record, team leaders record, supervisors report, a weekly report, and a spraying completion report. By 2005, a computerized data base developed by Booman et al. was adopted [18].

In this case, the GIS-based DSS has not only streamlined evidence-based implementation of interventions, but has improved the tracking of entomological indicators: species characterization and insecticide resistance status, including parasite prevalence and impact assessment of ITNs and IRS. It has been greatly valuable in enabling the display of heterogeneities in malaria risk areas within low transmission intensities [42, 46, 47]. The marked insecticide resistance problem in IRS (Mufweshya, Kabulongo, Kafue Estates, and Mukobeko) and LLIN (Rufunsa, Myooye, Chipepo, Chibombo, and Chiawa) deploying sites (Figure 2), confirms other findings of resistance developing in the wake of extensive vector control [48–50]. This allows the malaria control programme manager to better utilize the limited resources on insecticides to which the malaria vectors are still susceptible. Detection of high resistance levels has facilitated the planning of rational insecticide resistance management strategies and introduction of alternative noninsecticide-based vector control interventions. Due to low levels of transmission, malaria vector control interventions amenable to focalized implementation, such as larval source management using larvicides [51, 52] in the context of integrated vector management [13, 14], are being implemented.

The impact of main thrust vector control interventions on parasite prevalence in children between 1–14 years of age has been monitored through annual malaria surveys for three consecutive years at 19 sentinel sites (Figure 2). The use of the GIS-based DSS has facilitated for the assessment of the efficacy of IRS and ITNs either in combination or singly (Figure 6). In areas with high parasitaemia, this has allowed for the identification of areas that require replenishment of torn nets or areas that may require IRS instead of ITNs. Therefore, the value of any surveillance system for infectious disease is measured by its ability to provide timely, accurate “data for action” to people responsible for effective prevention and control activities and its ability to provide ongoing feedback to the primary gatherers of information [53, 54].

Although routine surveillance data have proved inadequate for monitoring control programmes [55], and have presently been supplanted by parasite prevalence surveys, vector-borne diseases demonstrate decided geographical heterogeneities and therefore require special tools for analysis [56]. The GIS with an inherent ability to manage spatial data provides an exceptional tool for continuous surveillance [57, 58] and provides a framework for harmonizing surveillance data and parasitaemia survey data. At a regional level, the ability of GIS to display data in an intuitively understandable manner has been harnessed to establish a continental database in Africa of spatial distribution of malaria [59] The DSS has been used to collate data on insecticide resistance in Africa [20].

The ability of GIS-based DSS to deal with large data sets and to incorporate satellite images increases the feasibility of studying transmission determinants of malaria and has resulted in prompt availability of data to support surveillance and policy formulation. The epidemiological mapping of high-risk areas of malaria transmission and insecticide resistance profiles of major vectors has facilitated the recognition of those populations and geographic areas where it is possible to identify the main determinants of malaria morbidity and mortality. The revealed trends and interrelationships have allowed the identification of high risk areas and facilitated decision making and rational utilization of limited resources in a cost-effective manner.

## 5. Conclusion

In Zambia, an evidence-based decision support has created a more focused and purposeful approach to directing resources to areas of most need with reasonable returns for effort and resources invested. Monitoring the impact of malaria vector control interventions through the GIS-based DSS on relative change in prevalence of infection and vector susceptibility to insecticides over time has enabled measurement of spatial heterogeneity of trend or impact. The revealed trends and interrelationships have allowed the identification of areas with reduced parasitaemia and increased insecticide resistance thus demonstrating the impact of vector control. Targeting interventions based on entomological and epidemiological evidence have not only contributed markedly to the success of the Zambian Malaria Control Programme, but also have provided opportunity for rational decision making in deployment of interventions and cost effective utilization of limited resources for enhanced malaria control.
